# Extracellular Matrix-Induced GM-CSF and Hypoxia Promote Immune Control of *Mycobacterium tuberculosis* in Human *In Vitro* Granulomas

**DOI:** 10.3389/fimmu.2021.727508

**Published:** 2021-09-17

**Authors:** Ainhoa Arbués, Sarah Schmidiger, Michael Kammüller, Damien Portevin

**Affiliations:** ^1^Department of Medical Parasitology and Infection Biology, Swiss Tropical and Public Health Institute, Basel, Switzerland; ^2^University of Basel, Basel, Switzerland; ^3^Translational Medicine-Preclinical Safety, Novartis Institutes for Biomedical Research, Basel, Switzerland

**Keywords:** tuberculosis, granuloma, extracellular matrix, GM-CSF (granulocyte-macrophage colony-stimulating factor), hypoxia, *in vitro* model

## Abstract

Several *in vitro* cellular models have been developed with the aim to reproduce and dissect human granulomatous responses, the hallmark of tuberculosis (TB) immunopathogenesis. In that context, we compared two- (2D) *versus* three-dimensional (3D) granuloma models resulting from infection of human peripheral blood mononuclear cells with *M. tuberculosis* (*Mtb*) in the absence or presence of a collagen-based extracellular matrix (ECM). Granuloma formation was found to be significantly enhanced in the 2D model. This feature was associated with an earlier chemokine production and lymphocyte activation, but also a significantly increased bacterial burden. Remarkably, the reduction in *Mtb* burden in the 3D model correlated with an increase in GM-CSF production. GM-CSF, which is known to promote macrophage survival, was found to be inherently induced by the ECM. We observed that only 3D *in vitro* granulomas led to the accumulation of lipid inclusions within *Mtb.* Our data suggest that a hypoxic environment within the ECM could be responsible for this dormant-like *Mtb* phenotype. Furthermore, exposure to a TNF-α antagonist reverted *Mtb* dormancy, thereby mimicking the reactivation of TB observed in rheumatic patients receiving this therapy. To conclude, we showed that only *in vitro* granulomas generated in the presence of an ECM could recapitulate some clinically relevant features of granulomatous responses in TB. As such, this model constitutes a highly valuable tool to study the interplay between immunity and *Mtb* stress responses as well as to evaluate novel treatment strategies.

## 1 Introduction

The appearance of granulomas is the hallmark of tuberculosis (TB), an ancient disease caused by *Mycobacterium tuberculosis* (*Mtb*) that still kills more than one million people every year (1). Granulomas are structurally organized cell aggregates composed of a core of macrophages in various differentiation states surrounded by a lymphocyte cuff (2). These structures constitute the landscape of interaction of the host’s immune system with *Mtb*. Therefore, a better understanding of granuloma biology could assist in the design of effective vaccination strategies and/or host-directed therapies that are urgently needed to improve the management of the TB epidemic.

For ethical reasons, investigations on human granulomas cannot be performed in patients. Even when lung resection samples are available, these structures provide only a static image of the latest stages of the disease. Therefore, animal models have helped to generate a dynamic picture of the immunological processes involved in the genesis and evolution of granulomatous structures (2). However, *Mtb sensu stricto* only infects humans and some of the immune pathways involved may therefore diverge from those observed in animals (3, 4). The intent to overcome these limitations resulted in the development of several *in vitro* granuloma models over the past fifteen years (5). Most commonly used models are based on the stimulation of human peripheral blood mononuclear cells (PBMCs), which have the advantage of being easily accessible. In the early days, PBMCs were cultured in flat bottom plates, which offer a bi-dimensional (2D) environment, and the formation of cell aggregates was induced by addition of antigen-coated agarose beads (6). Soon those beads were substituted by *Mtb* bacilli (7, 8), thereby increasing the meaningfulness of these models. The subsequent incorporation of an extracellular matrix (ECM) rendered the *in vitro* models more physiological and added a third dimension (3D). Elkington’s group made use of bio-electrospray technology to generate alginate-collagen microspheres within which the *in vitro* granulomas develop (9). This 3D microsphere model demonstrated interesting potential; yet, the specialized equipment and expertise required constitutes an obstacle for the implementation of this model in other laboratories. Kapoor et al. (10) implemented yet another 3D *in vitro* granuloma model by embedding the infected PBMCs within a fibronectin and collagen ECM in a microplate format. Interestingly, this model exhibited the remarkable ability to induce dormant-like features in the tuberculous bacilli. Collectively, these models have contributed to enhance our understanding of the mechanisms that regulate host-pathogen interactions in human granulomas. They have also proven their worth as tools to evaluate the activity of anti-TB drugs (11) and the risk of certain immunotherapies to reactivate latent TB (10, 12, 13). Both, 2D and 3D *in vitro* models are still readily used in different laboratories to investigate human TB immunology. However, to our knowledge, 2D and 3D models have never been compared systematically to assess whether and how they differ in capturing relevant features of granulomatous responses observed *in vivo*.

To fill this gap, we aimed to compare the performance of microplate-based, 2D and 3D granuloma models side by side, by following up relevant immunological and microbiological readouts. We observed that the formation of cell aggregates together with the activation of an adaptive immune response are favored in the absence of an ECM. By contrast, the ECM promotes a better survival of macrophages and delays *Mtb* growth, most likely *via* an earlier induction of GM-CSF secretion. Furthermore, we demonstrate that the presence of an ECM creates a hypoxic environment that can prompt *Mtb* to acquire a dormant-like phenotype.

## 2 Material and Methods

### 2.1 Preparation of Human Peripheral Blood Mononuclear Cells

Buffy coats from healthy blood donors (under informed consent) were purchased from the Interregionale Blutspende SRK AG (Bern, Switzerland). PBMCs were isolated by Ficoll-Paque (GE Healthcare) density-gradient centrifugation and washed twice in RPMI-1640 with L-glutamine (RPMI; Sigma-Aldrich). Aliquots were cryopreserved in RPMI containing 10% DMSO (Sigma-Aldrich) and 40% fetal bovine serum (FBS; Gibco) and stored in liquid nitrogen until use. Prior to use, PBMCs were systematically tested for CD4 T cell memory reactivity against Purified Protein Derivative (PPD) from *Mtb* as previously reported by our laboratory (12). When needed, PBMCs from various PPD-reactive donors (**Table S1**) were thawed, washed twice in RPMI containing 10% FBS and benzonase (12.5 U/ml; BioVision) and rested in RPMI supplemented with 10% FBS at 37°C (5% CO_2_) overnight. Trypan blue dye exclusion method was used to confirm sample viability of above 95%. PBMC concentration was adjusted to 10^7^ cells/ml in RPMI supplemented with 20% human serum (PAN-Biotech) (referred to as “cell culture medium” from here on).

### 2.2 Generation of Disperse *Mtb* Suspensions

*Mtb* H37Rv and H37Ra were cultured in Middlebrook 7H9 broth supplemented with 10% ADC (5% bovine albumin fraction V, 2% dextrose and 0.003% catalase), 0.5% glycerol (PanReac AppliChem) and 0.1% Tween-80 (Sigma-Aldrich). Upon reaching mid-exponential phase, bacteria were washed with PBS containing 0.1% Tween-80 (PBST) and re-suspended in cell culture medium. The resulting *Mtb* suspension was dispersed by water-bath sonication (XUB5, Grant Instruments) for 2 min, and then centrifuged at 260 × g for 5 min. The upper part of the supernatant (containing mostly single bacteria) was recovered and cryopreserved by adding 5% glycerol (final) and storing at –80°C. Concentration of the frozen stocks was quantified by colony forming unit (CFU) assessment. Briefly, 10-fold serial dilutions were prepared in triplicate in PBST and plated on Middlebrook 7H11 agar plates supplemented with 0.5% glycerol and 10% OADC (0.05% oleic acid in ADC). Plates were incubated at 37°C (5% CO2) for 3-4 weeks.

### 2.3 *In Vitro* Granuloma Models

Rested PBMCs were infected with *Mtb* at a multiplicity of infection (MOI) of 1:200 (*Mtb :* PBMC), or left uninfected when required, then split in half to be processed according to the pertinent granuloma model.

#### 2.3.1 Two-Dimensional Model

Infected PBMCs were topped up with cell culture medium to reach a final concentration of 2.5 × 10^6^ cells/ml and 1 ml per well was distributed across 24-well plates.

#### 2.3.2 Three-Dimensional Model

2.5 × 10^6^ infected PBMCs (250 μl) per well were distributed across 24-well plates. Thereafter, cells were embedded within an extracellular matrix (ECM) by adding 250 μl per well of a mixture composed of 950 μl/ml of collagen (3.1 mg/ml; PureCol collagen solution, Advanced BioMatrix), 50 μl/ml of PBS 10X (Sigma-Aldrich), 4 µl/ml of fibronectin (1 mg/ml; Sigma-Aldrich) and 10 μl of 1N NaOH (Sigma-Aldrich). The ECM was left to set for 45 min at 37°C (5% CO_2_) before 500 μl of cell culture medium were added.

Plates were incubated at 37°C (5% CO_2_) and granuloma formation was monitored on days 1, 4 and 8 post-infection using a Leica DM IL LED inverted microscope and a Leica MC170 HD camera. The areas encompassing cellular aggregates were scored in ImageJ 1.52n (National Institutes of Health).

On day 4 post-infection, if applicable, 250 μl of supernatant were replaced by the same volume of fresh cell culture medium containing adalimumab (Humira, Abvie) – a humanized anti-human TNF-α antibody – or a relevant isotype control (human IgG1, clone ET901; Biolegend) at a final concentration of 10 ng/ml.

To study the impact of GM-CSF on bacterial load, 2D granulomas were treated with recombinant human GM-CSF (Peprotech) at a final concentration of 5 ng/ml two hours post-infection.

### 2.4 Retrieval of *Mtb*

At the relevant time points, 250 μl of supernatant were removed from the 2D model wells, whereas the supernatant was removed completely in the 3D model and the ECM was digested with 250 μl of collagenase (1 mg/ml; Sigma-Aldrich) for 40 min at 37°C (5% CO_2_). For both models, PBMCs were subsequently lysed by adding 250 μl of 0.4% Triton X-100 (Sigma-Aldrich) and incubating for 20 min at room temperature. Released bacilli were used for bacterial load quantification by CFU assessment and for dual auramine-O/Nile red staining.

### 2.5 Dual Auramine-O/Nile Red Staining

*Mtb* retrieved from both granuloma models were pelleted by centrifugation at 6000 × g for 5 min prior to being inactivated with 1X CellFIX (BD Biosciences) for 20 min at room temperature. Fixed samples were spotted on glass slides, air dried and heat fixed at 70°C for at least 2 h. Acid-fast staining using TB Fluorescent Stain Kit M (BD) was performed in combination with neutral lipid staining dye Nile red (Sigma-Aldrich). Each sample was stained with auramine-O for 20 min, decolorized for 30 s, covered with Nile red (10 μg/ml in ethanol) for 15 min and counterstained with potassium permanganate for 2 min. Samples were gently washed with distilled water between each step. Stained slides were examined using a Leica DM5000 B fluorescence microscope. For quantitative analysis, at least 200 bacteria per sample were counted.

### 2.6 Flow Cytometry Analysis

PBMCs were recovered from both granuloma models at the relevant time points as follows. In the 3D model, PBMCs were released from the ECM as described above. In the 2D model, suspension cells were recovered by pipetting up and down and transferred to a tube. Afterwards, remaining adherent cells were detached by treatment with 250 μl of accutase (Sigma-Aldrich) for 30 min at 37°C (5% CO_2_), and subsequently pooled with the suspension cells. In both cases, cells were pelleted at 400 × g for 5 min, washed once with FACS buffer (PBS containing 1% FBS), and then extracellularly stained following a standard protocol. Cells were incubated for 20 min at room temperature in 50 μl of cold FACS buffer containing fluorochrome-labeled antibodies against human CD3 (VioBlue, clone REA613; Miltenyi Biotec), CD4 (VioBlue, clone REA623; Miltenyi Biotec), CD8 (PE-Cy7, clone HIT8a; Biolegend), CD19 (BV510, clone HIB19; Biolegend), CD56 (APC-Cy7, clone REA196; Miltenyi Biotec), CD40 (FITC, clone 5C3; Biolegend), CD206 (PE, clone 15-2; Biolegend), CD69 (PerCP, clone FN50; Biolegend), and HLA-DR (APC, clone L243; Biolegend). Samples were washed once with FACS buffer and fixed in 1X CellFIX for 20 min at room temperature. Samples were acquired on a MACSQuant Analyzer 10 (Miltenyi Biotec) and processed using FlowJo 10.6.1 (BD).

### 2.7 Macrophage Phagocytic Activity and ROS Production

To investigate phagocytic activity and ROS production of macrophages recovered from 2D *versus* 3D granulomas, uninfected PBMCs were processed according to both granuloma models in a 24-well plate format. On day 4 post-infection, PBMCs were retrieved as described in section 2.6. Recovered PBMCs were washed once in RPMI, re-suspended in cell culture medium and counted by Trypan blue exclusion method.

#### 2.7.1 Phagocytic Activity

Recovered PBMCs were transferred to tubes for subsequent infection with H37Ra::GFP at MOIs 1:10 and 1:1 (*Mtb :* PBMC) for two hours at 37°C (5% CO_2_). Uninfected controls were processed in parallel. After the infection, cells were washed once with FACS buffer and extracellularly stained with fluorochrome-labeled antibodies against human CD33 (APC-Cy7, clone WM53; Biolegend), HLA-DR (APC, clone L243; Biolegend) and CD206 (BV510, clone 15-2; Biolegend) for 15 min on ice in the dark. Cells were washed, fixed and acquired as described above.

#### 2.7.2 Reactive Oxygen Species Production

Recovered PBMCs were transferred to tubes for subsequent infection with H37Ra at an MOI of 1:1 (*Mtb :* PBMC) for two hours at 37°C (5% CO_2_). Uninfected controls for both models were processed in parallel. Additionally, an unstained control (2D model) was included. After the infection, cells were washed once in PBS and re-suspended in 100 µl of PBS containing 10 µl of ROS probe (ROS-ID hypoxia/oxidative stress detection kit, Enzo Life Sciences) and 1 µl of anti-human CD33 antibody (APC-Cy7, clone WM53; Biolegend). The unstained control was re-suspended in 100 µl FACS buffer and stained with anti-CD33 only. All samples were incubated at 37°C (5% CO_2_) for 30 min before being washed once with PBS. Samples were fixed and acquired as described above.

### 2.8 Detection of Hypoxia

To assess hypoxia induction, *in vitro* granulomas were generated with *Mtb* H37Rv or H37Ra in a 96-well plate format. On day 7 post-infection, PBMCs were recovered and washed as described in the previous section. Uninfected PBMCs cultured under standard conditions (2D) were included as a “normoxic” control. The level of hypoxia was evaluated using ROS-ID Hypoxia/Oxidative Stress Detection Kit (Enzo Life Sciences) according to the manufacturer’s recommendations. In short, cells were stained in 100 μl of diluted Hypoxia Detection Reagent (1:1000 in PBS) for 30 min at 37°C (5% CO_2_), washed once with PBS and fixed in 1X CellFIX for 20 min at room temperature. Samples were acquired on a MACSQuant Analyzer 10 (Miltenyi Biotec) and processed using FlowJo 10.6.1 (BD).

### 2.9 Cytokine Quantification

Supernatants collected at the relevant time points and stored at –80°C were filter-sterilized no more than 24 h prior to analysis by multiplex bead-based immunoassay. Immune Monitoring 65-Plex Human ProcartaPlex Panel (Invitrogen) was used according to the manufacturer’s recommendations and acquired on a Luminex Bio-Plex 200 platform and Bio-Plex Manager 6.0 software (Bio-Rad). Culture medium was measured in parallel to the samples to assess any potential residual presence of the target biomolecules in the human serum. Results were analyzed using R package nCal.

### 2.10 Quantification and Statistical Analysis

GraphPad Prism 8.2.1 and R.4.0.3 were used to produce quantitative graphical representations of the generated data and to perform statistical analysis. The number of donors used, definition of center and dispersion measures, and nature of the tests are specified within the respective figure legend.

## 3 Results

### 3.1 Extracellular Matrix Delays the Development of *In Vitro* Granulomas

**Figure 1:**
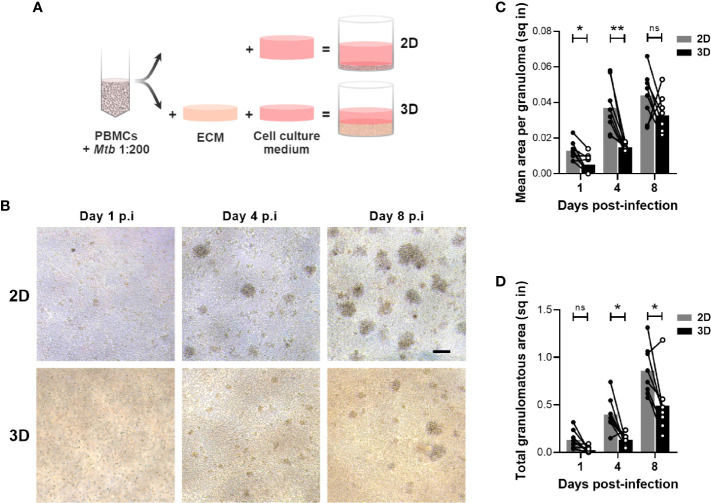
Development of *in vitro* granulomas is delayed in the 3D model. **(A)** Schematic representation of the side-by-side experimental setup. ECM, extracellular matrix. **(B)** Bright field images of *in vitro* granulomas formed in the absence (2D) or presence (3D) of an ECM from a representative donor, viewed under 10X magnification. Scale bar = 100 μm. **(C, D)** Quantification of mean granuloma size **(C)** and total granulomatous area **(D)**. Circles represent the area in square inches (sq in) of all cell aggregates observed in a representative image for each individual donor, lines connect results from the same donor, and bars indicate mean values of eight donors. Statistical analysis was performed using two-way repeated measures ANOVA and Sidak’s multiple comparisons test. ns, non-significant; *p < 0.05; **p < 0.01.

Various *in vitro* granuloma models, encompassing (3D) or not (2D) an extracellular matrix (ECM), have been developed to investigate the interaction between human immune cells and *Mtb* (8–10, 12, 14). Yet, to our knowledge, the performance of 2D and 3D granuloma models has never been compared. Intending that, we ran both models in a side-by-side experimental setup (**Figure 1A**). PBMCs isolated from the blood of healthy donors were infected with *Mtb* H37Rv and subsequently split to be embedded, or not, in an ECM composed of collagen and fibronectin. We first evaluated the capacity of these two models to allow the formation of *Mtb-*induced cellular aggregates. Uninfected PBMCs were included as control for spontaneous aggregation, and no significant aggregation was observed at any of the studied time points. A representative bright field was imaged and granuloma number and size were evaluated at the indicated time points for each PBMC donor (n=8) (**Figure 1B**). Image quantification showed that cellular aggregates formed faster and were significantly bigger in the absence of an ECM (**Figure 1C**). While small cell clusters were detected after one day in the absence of an ECM, aggregates of a similar size were only detected 4 days post-infection in presence of an ECM. Consequently, the total granulomatous area was increased by approximately twofold in the 2D model (**Figure 1D**). This increase reflects the presence of bigger and/or more numerous aggregates in the absence of an ECM.

**Figure 2:**
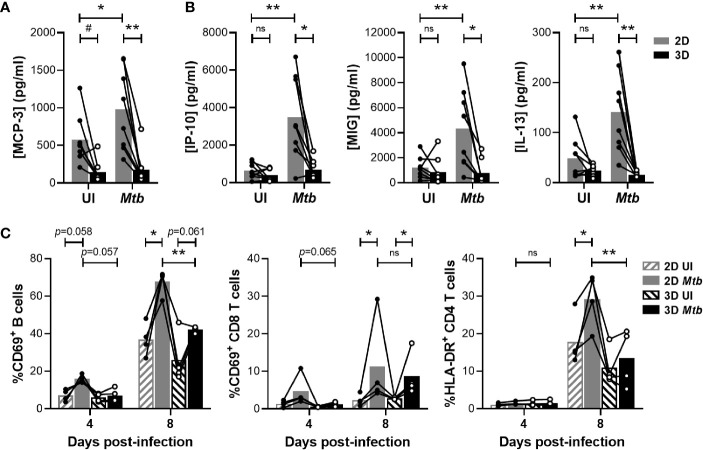
Embedding within an ECM hampers chemokine secretion and lymphocyte activation. **(A, B)** Concentration of MCP-3 on day 1 **(A)** and IP-10, MIG and IL-13 on day 4 **(B)** quantified by multiplex bead-based immunoassay. UI, uninfected. **(C)** Proportion of activated B cells and CD4 or CD8 T cells determined by flow cytometry. Circles represent the value for each individual donor, lines connect results from the same donor, and bars indicate mean values of eight **(A, B)** or four **(C)** donors. Statistical analysis was performed using two-way repeated measures ANOVA and Sidak’s multiple comparisons test. ns, non-significant; *p < 0.05; **p < 0.01; #, no longer significant upon correction for multiple cytokine analysis.

The formation of aggregates relies on cell recruitment following a chemoattractant gradient. Therefore, we evaluated whether the two models induced distinct chemokine levels that could account for the differences observed in granuloma formation. A bead-based multiplex assay was used to quantify the accumulation of 65 soluble factors in the supernatant of the *in vitro* granulomas. A comparative analysis revealed a group of cytokines that were detected earlier in the 2D model (**Figure S1**, depicted in blue). In line with the faster aggregate formation, higher levels of Monocyte Chemoattractant Protein 3 (MCP-3) were induced in the absence of an ECM on day 1 (**Figure 2A**). This difference was further increased upon infection with *Mtb*. Moreover, on day 4 post-infection, IFN-γ-induced pro-inflammatory chemokines IP-10 and MIG were detected in the supernatant of 2D but not yet in that of 3D granulomas (**Figure 2B**). A potential interaction of the ECM with chemoattractant proteins (15) leading to an apparent decrease in their diffusion/availability could contribute to this observation. A similar pattern was observed for the Th2 cytokine IL-13. The delayed adaptive-cytokine response and formation of aggregates in the presence of an ECM suggested a differential activation of lymphocytes. To assess this, all cells present in the wells were harvested in the course of granuloma formation, and the expression of activation markers CD69 and HLA-DR was analyzed on the lymphocyte subsets by flow cytometry (**Figure S2**). The proportion of CD69^+^ B cells and CD8 T cells was lower on day 4 post-infection in the 3D compared to the 2D model (**Figure 2C**). By day 8, this difference had waned for the CD8 T cells, while it became significant for the B cells. CD4 T cell activation was also significantly lower in the 3D model on day 8 post-infection. Taken together, the presence of an ECM impedes granuloma formation most likely through a delayed activation of lymphocytes and release of chemotactic factors.

### 3.2 ECM-induced GM-CSF Promotes Survival of Macrophages and Control of *Mtb* Proliferation

**Figure 3:**
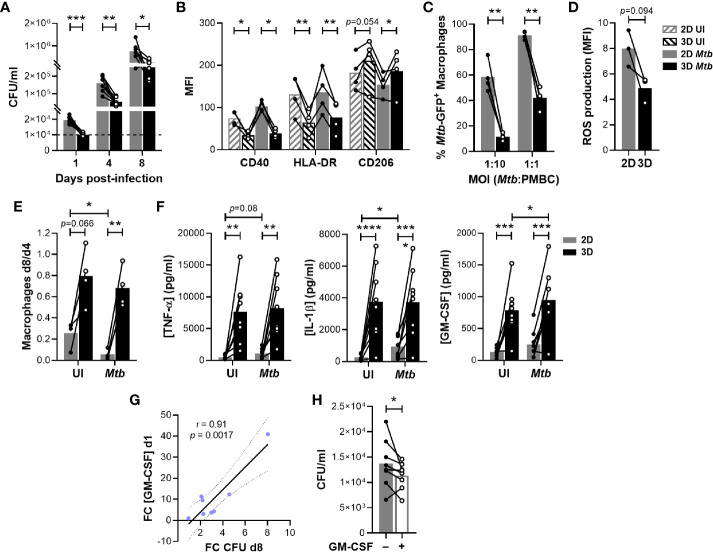
The ECM hinders *Mtb* growth and enhances macrophage survival by inducing GM-CSF. **(A)** Quantification of bacterial load by colony forming unit (CFU) assessment. The dashed line indicates the inoculum. **(B)** Flow cytometry analysis of the expression of macrophage polarization markers CD40, HLA-DR and CD206 on day 4. MFI, median fluorescence intensity; UI, uninfected. **(C)** Flow cytometry analysis of the phagocytic capacity of macrophages retrieved on day 4 and subsequently infected with *Mtb* H37Ra::GFP. **(D)** Flow cytometry analysis of ROS production by macrophages retrieved on day 4 and subsequently infected with H37Ra (MOI 1:1). **(E)** Macrophage survival estimated by flow cytometry as the ratio of macrophages remaining on day 8 compared those present on day 4. **(F)** Concentration of TNF-α, IL-1β and GM-CSF in the supernatants on day 1 quantified by multiplex bead-based immunoassay. **(G)** ECM-induced increase on GM-CSF concentration on day 1 correlates with the decrease in CFU observed on day 8. FC, fold change. Data were analyzed using Pearson’s correlation test. Grey dotted lines represent 95% confidence intervals. **(H)** Quantification by CFU assessment of bacterial load in 2D granulomas after one day of infection in the absence or presence of exogenous GM-CSF (5 ng/ml). **(A–F, H)** Circles represent the value for each individual donor, lines connect results from the same donor, and bars indicate mean values of eight **(A, F)**, four **(B, C, E)** or three **(D)** donors. Statistical analysis was performed using two-way repeated measures ANOVA and Sidak’s multiple comparisons test **(A–C, E, F)** or paired t test **(D, H)**. *p < 0.05; **p < 0.01; ***p < 0.001; ****p < 0.0001.

Having evidenced a delayed granulomatous response in the presence of ECM, we investigated whether this would impact bacterial replication. As represented in **Figure 3A**, *Mtb* proliferated in both models throughout the course of the assay. Strikingly, bacterial burden was significantly lower in the presence of ECM at all investigated time points. In fact, in the 2D model CFUs were already increased by a factor of 1.98 ± 0.39 (Mean ± SD) one day post-infection. Therefore, we wondered whether the presence of the ECM possibly impeded the growth of non-phagocytosed extracellular bacilli. To rule out this possibility, we ran both protocols using the same amount of bacteria but without PBMCs. *Mtb* growth could not be observed in cell culture medium regardless of the presence or absence of ECM (**Figure S3**). Instead, the bacterial load had substantially decreased by day 8, suggesting that *Mtb* requires the presence of host cells to replicate and survive.

Our results suggested that host cells are better equipped to control mycobacterial replication in the presence of an ECM at the earliest stages of the infection. We hypothesized that this could be linked to the polarization of macrophages. Classically activated macrophages (M1) display a boosted microbicidal phenotype, whereas M2 macrophages exhibit anti-inflammatory properties. To evaluate the polarization state of macrophages in both granuloma models, we assessed relevant macrophage surface markers by flow cytometry on day 4 post-infection (**Figure S4**). Independently of infection, expression of M1-like markers CD40 and HLA-DR was significantly increased on macrophages retrieved from the 2D model (**Figure 3B**). In turn, M2 marker CD206 expression tended to be more pronounced in the 3D model. We then aimed to functionally confirm the enhanced M2 phenotype of macrophages retrieved from the 3D model. To do so, we investigated the capacity of macrophages generated in the absence or presence of an ECM to phagocytose and produce reactive oxygen species (ROS). Consistent with an M2 profile, macrophages retrieved from a 3D environment displayed significantly reduced phagocytic activity (**Figure 3C**) and showed a tendency for reduced ROS production (**Figure 3D**). Taken together, the presence of an ECM was consistently associated with an M2-like phenotype known to display a low antimicrobial capacity. Thus, differential macrophage polarization cannot explain the decreased bacterial load observed in the 3D model. At the same time, the embedding within an ECM significantly improved macrophage survival on day 8, independently of infection (**Figure 3E**). Therefore, we assessed whether the ECM might trigger the release of soluble factors that could promote cell survival and/or delay *Mtb* growth. Indeed, we observed a cluster of cytokines and growth factors preferentially secreted by ECM-embedded host cells at the earliest time point, even when they were uninfected (**Figure S1**, depicted in red). This cluster encompassed the pro-inflammatory cytokines IL-1β, TNF-α and GM-CSF (**Figure 3F**). Since large amounts of TNF-α can lead to cell death (16), we verified by flow cytometry that the cell numbers of T and B cell populations were comparable between both models (**Figure S6**). Remarkably, only the increase in concentration of GM-CSF in 3D compared to 2D correlated with the difference in bacterial load observed on day 8 (**Figure 3G**). In fact, the addition of exogenous GM-CSF to 2D granulomas ameliorated control of *Mtb* within one day of infection (**Figure 3H**). Altogether, these results suggest that ECM-induced secretion of GM-CSF promotes macrophage survival and increases control of *Mtb* growth within human granulomas.

### 3.3 Hypoxia Induced by the ECM Drives *Mtb* into a Dormant-Like State

**Figure 4:**
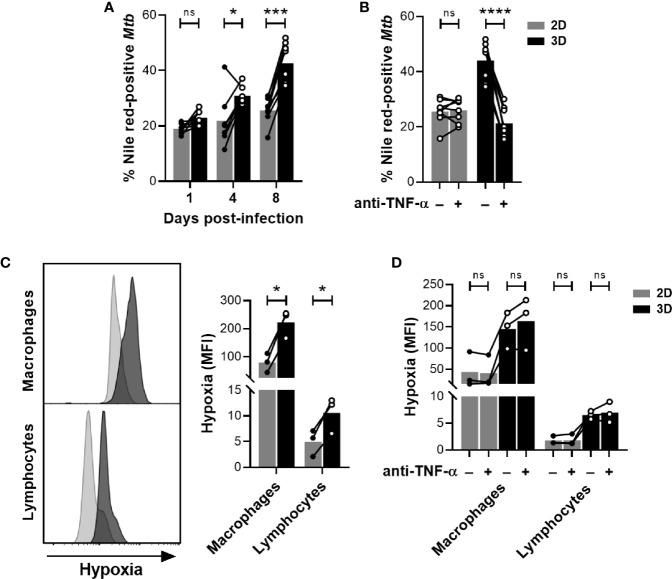
The presence of an ECM induces hypoxia and promotes *Mtb* dormancy. **(A, B)** Proportion of dormant-like *Mtb* quantified by fluorescence microscopy upon dual staining with auramine-O and Nile red. **(B)** On day 4 post-infection, *in vitro* granulomas were treated with the anti-TNF-α antibody adalimumab (+) or an isotype control (–). Proportion of dormant-like *Mtb* was quantified after four days of treatment (day 8 post-infection). **(C)** On day 7 post-infection, hypoxia was evaluated by flow cytometry using a fluorescent probe. **(D)** On day 4 post-infection, *in vitro* granulomas were treated with the anti-TNF-α antibody adalimumab (+) or an isotype control (–). Flow cytometry analysis of hypoxia using a fluorescent probe was performed after four days of treatment (day 8 post-infection). Circles represent the value for each individual donor, lines connect results from the same donor, and bars indicate mean values of eight **(A, B)** or three **(C, D)** donors. Statistical analysis was performed using two-way repeated measures ANOVA and Sidak’s multiple comparisons test. ns, non-significant; *p < 0.05; ***p < 0.001; ****p < 0.0001.

We, and others, previously demonstrated that *in vitro* granulomas generated in the presence of an ECM trigger a mycobacterial metabolism switch into dormancy (10, 12). Thereby, 3D granuloma models have been validated as useful tools to assess the risk of latent TB reactivation potentially associated with certain biologic therapies (10, 12, 13). However, to our knowledge, whether dormancy was also induced in the absence of an ECM had never been assessed. Among other phenotypes, dormant bacilli lose their acid-fastness and accumulate triacylglycerides, which can be stained with the fluorescent dye Nile red. To compare dormancy induction between both models, we subjected bacteria recovered from both granuloma models to an auramine-O/Nile red dual staining. The proportion of Nile red-positive bacilli that have lost their acid fastness (auramine-O negative) was used as a proxy for dormancy induction. Granulomas generated in the presence of an ECM displayed a significantly higher proportion of Nile red-stained bacilli on day 4, which was further increased four days later (**Figure 4A**). Nonetheless, a minor accumulation of Nile red-positive *Mtb* could also be observed in the absence of an ECM between days 4 and 8 post-infection. To test whether the 2D model could also be exploited for TB reactivation risk assessment, we used adalimumab, a humanized anti-human TNF-α antibody known to induce *Mtb* resuscitation (12), as a positive control. As represented in **Figure 4B**, anti-TNF-α treatment promoted *Mtb* resuscitation (measured as a decrease in the proportion of Nile red-positive bacilli) in the presence of an ECM. Contrarily, adalimumab failed to revert the minor accumulation of dormant-like *Mtb* observed in the 2D model.

The sensing of a hypoxic environment is one of the stresses that can push *Mtb* into a dormant state (17). This phenomenon is of particular relevance in the context of 3D granuloma models since incorporation of ECMs in a cell culture can diminish oxygen tension (18). In order to evaluate a potential limitation in oxygen availability, we took advantage of a chemical probe whose fluorescence depends on the nitroreductase activity present in hypoxic cells. We quantified the intensity of the hypoxia probe by flow cytometry in morphologically gated macrophages and lymphocytes. A significant increase in fluorescence was detected in 3D compared to 2D granulomas for both cell populations, proving that the presence of an ECM leads to a more pronounced hypoxic environment (**Figure 4C**). Importantly, the induction of a hypoxic environment was not reverted upon anti-TNF-α treatment (**Figure 4D**). Therefore, our results suggest that the presence of an ECM induces *Mtb* dormancy in a hypoxia-dependent manner.

## 4 Discussion

*Mtb* infection triggers a spatially-organized immune response in the lungs of TB patients leading to the formation of a spectrum of characteristic granulomas (19). On the one hand, granulomatous responses may succeed in encapsulating the infection foci and even sterilizing them. On the other hand, excessive inflammation, cellular recruitment and necrosis can lead to granuloma liquefaction. Ensuing tissue destruction may eventually give rise to cavities which substantially increase morbidity even when TB has been cured. We do not yet understand the components of protective immunity required to elicit sterilization within human TB granulomas. Development of novel TB treatments and vaccine strategies would benefit from a better understanding of the interaction between the various components of the human immune system and *Mtb* within these structures. Non-human primates undoubtedly constitute the most relevant animal model for the study of granuloma biology (20). However, the high costs and ethical restrictions substantially hamper their systematic use. Consequently, various PBMC-based *in vitro* models were developed to study the host-pathogen interactions within human granulomas (5). Being based on easily accessible human primary immune cells, *in vitro* granuloma models are particularly relevant and cost-effective. While an ECM is incorporated in 3D models to further increase their physiological relevance, in other models *in vitro* infection of PBMCs is performed in two dimensions. Infection of PBMCs in 2D substantially increase the possibility to perform high-throughput screening experiments since the embedding within an ECM makes 3D models technically more challenging and time-consuming. To demonstrate the advantages and limitations of the different models, side-by-side evaluation of 2D and 3D granuloma models became necessary.

In this report, we decided to compare microplate format-based models that do not require any specialized equipment and could easily be implemented within any biosafety level 3 laboratory. When we compared the two types of models side by side, we observed a faster cell recruitment and activation in the absence of an ECM. At first glance, the faster and bigger granuloma formation in the 2D model intuitively suggested that the strength of these immune reactions would translate to a better control of *Mtb* replication. Nonetheless, a lower bacterial burden was detected in the 3D model at all the investigated time points. In addition, the presence of an ECM improved the survival of macrophages upon infection. This superior, ECM-dependent macrophage survival could have been a consequence of the lower bacterial load building up in the 3D model. However, the fact that a similar trend was observed in the absence of *Mtb* infection hinted at a bacterial load-independent phenomenon. Our results actually corroborate those of Elkington’s group showing that the addition of collagen favors the host over *Mtb* (9, 21). Still, the underlying mechanisms sustaining these differential phenotypes were not yet elucidated.

We found that the incorporation of the ECM significantly increased the concentration of TNF-α, IL-1β and GM-CSF in the supernatants at the earliest time point. Interestingly, the expression of these three pro-inflammatory cytokines is linked, and they constitute an inflammatory network that contributes to the pathology of inflammatory and autoimmune syndromes such as arthritis, experimental autoimmune encephalomyelitis (EAE) and cardiovascular or lung diseases (22). Regarding the origin of these cytokines, several studies have described that the interaction of monocytes with the ECM components collagen and fibronectin induces the secretion of IL-1β and TNF-α (23, 24). In turn, IL-1β and TNF-α induce the secretion of GM-CSF by non-hematopoietic cells, thereby promoting monocyte survival (25). While PBMC-based granuloma models lack non-hematopoietic cells, upregulation of GM-CSF has also been reported in monocytes upon adhesion to fibronectin (26). In line with their major role in inflammation, TNF-α and GM-CSF contribute to the control of *Mtb* infection (27, 28). Remarkably, among the different cytokines induced by the ECM, only GM-CSF showed a correlation with the containment of *Mtb* growth in the 3D model. Our finding contrasts a previously observed one, where addition of exogenous GM-CSF resulted in a higher bacterial load (13). This divergence could be explained by a detrimental effect of an excess of GM-CSF. Dose-response experiments would have to be carried out to ascertain this interpretation. However, such bimodal behavior has been described for TNF-α, where both neutralization (12) and excessive levels (13) lead to increased bacterial proliferation. Thus, our result reinforces the concept that a balanced inflammatory response may be critical to control TB.

Among other traits, *Mtb* may notably survive for long periods within host granulomas by entering into dormancy (29). The phenotypes associated with dormant bacilli are a reduced metabolic activity, an increased lipid metabolism and antibiotic tolerance, and the inability to grow on solid medium (“non-culturability”). The transcription factor DosR (DOrmancy Survival Regulator) coordinates the expression of specific genes required for this metabolic shift in response to stresses related to *Mtb* intracellular niche, such as oxidative stress as well as nutrient starvation and hypoxia, a characteristic of granulomatous lesions (17, 30). Previous reports, including ours, revealed that ECM-embedded granulomas promote *Mtb* entry into a dormant-like phenotype (10, 12). This phenotype is typified by the accumulation of intracytoplasmic lipid inclusions and the induction of DosR-regulated genes, similarly to what was described for mycobacteria retrieved from the sputum of TB patients (31). Importantly, we reported here that this relevant feature can only be reproduced in a normoxic incubator when granulomas are elicited in the presence of an ECM. Indeed, no significant accumulation of Nile red-positive *Mtb* was detected in the 2D model. Actually, the lower *Mtb* burden attained in the presence of ECM could be linked, at least partially, to this increase in dormant-like *Mtb* fraction, given that it would translate into a non-culturable population. Although this did not fall within the scope of this study, it would be interesting to apply limiting dilution in liquid culture – in the presence or not of resuscitation factors – to quantitatively assess the presence of non-culturable bacteria within *in vitro* granulomatous responses in the presence of an ECM (32).

Lastly, we demonstrated that ECM embedding subjects the cells to a hypoxic environment and hence promotes *Mtb* dormancy. Incorporation of an ECM into a cell culture can indeed lead to a drop of the oxygen tension (18). The existence of hypoxic regions within the granulomatous lesions has been demonstrated in TB patients (33) as well as in susceptible animal models (30). Hypoxia is one of the main signals inducing the expression of the DosR operon (17). The presented results demonstrate that only 3D *in vitro* granulomas manage to reproduce the hypoxic environment detected *in vivo*. Besides its effect on *Mtb* metabolism, hypoxia can also have an impact on the host cells. We observed a switch in macrophage polarization profiles retrieved from 2D *versus* 3D granulomatous responses. Interestingly, and consistent with our results, monocytes differentiated under hypoxic conditions showed a decrease in M1- while increasing the M2-associated markers (34). Furthermore, hypoxia has been reported to enhance macrophage survival in a GM-CSF-independent manner (35) that could also contribute to the favored survival observed in the presence of an ECM.

Despite displaying numerous interesting features, *in vitro* granuloma models based on PBMCs have important limitations that should be addressed in future studies. Firstly, the incorporation of other relevant cell types, such as neutrophils or non-hematopoietic cells, would be worthy to be pursued. Since TB is a chronic disease developing over months or years, prolongation of the experiments would additionally increase their relevance. Actually, the incorporation of a washing step after infection of PBMCs and prior embedding, similar to the procedure performed in the microsphere model (9), may allow to maintain the model for several weeks. While it would be of high interest, current approaches to retrieve cells from *in vitro* granuloma models hamper studying cells within individual aggregates. Finally, the lack of a continuous influx of fresh immune cells yet constitutes a challenging limitation to overcome that may require implementing a microfluidic-based culture system.

Altogether, we have shown that the ECM promotes host cell survival and *Mtb* control by inducing GM-CSF and hypoxia. Our findings strongly support the inclusion of an ECM to induce relevant features of human TB granulomas *in vitro* (5). In particular, the induction of hypoxia within 3D granulomatous responses constitutes a unique asset to study immunological and microbiological processes involved in *Mtb* dormancy and resuscitation. To fully exploit this feature of 3D granulomatous responses, it would be highly interesting to investigate the underlying mechanisms of hypoxia induction and the direct link to *Mtb* dormancy in our model. This is of particular interest in an era of active development of new biotherapeutics that may interfere with, but also contribute to, TB protective immunity.

## Data Availability Statement

The raw data supporting the conclusions of this article will be made available by the authors, without undue reservation.

## Ethics Statement

Ethical review and approval was not required for the study on human participants in accordance with the local legislation and institutional requirements. The patients/participants provided their written informed consent to participate in this study.

## Author Contributions

DP led the project. AA and DP designed the study. AA and SS carried out the experiments. AA, SS, and DP contributed to the analysis and interpretation of the results. AA took the lead on writing the draft of the manuscript. AA, SS, MK, and DP provided critical feedback and contributed to shape the final version of the manuscript. All authors contributed to the article and approved the submitted version.

## Funding

Funding supporting this study is gratefully acknowledged from the Swiss National Science Foundation (Project grant: 310030_197838). Some reagents had been previously obtained via funds from a research agreement contract between Novartis AG and the Swiss Tropical and Public Health Institute. DP has received a research grant from Novartis. The funder was not involved in the study design, collection, analysis, interpretation of data, the writing of this article or the decision to submit it for publication.

## Conflict of Interest

MK is a full-time employee of Novartis. The remaining authors declare that the research was conducted in the absence of any commercial or financial relationships that could be construed as a potential conflict of interest.

## Publisher’s Note

All claims expressed in this article are solely those of the authors and do not necessarily represent those of their affiliated organizations, or those of the publisher, the editors and the reviewers. Any product that may be evaluated in this article, or claim that may be made by its manufacturer, is not guaranteed or endorsed by the publisher.
