# Supplementary material for: Extracellular Matrix-Induced GM-CSF and Hypoxia Promote Immune Control of Mycobacterium tuberculosis in Human In Vitro Granulomas
Source: Front Immunol. 2021 Sep 17;12:727508. doi: 10.3389/fimmu.2021.727508 (PMC8486295; doi:10.3389/fimmu.2021.727508)
Supplement: Supplementary file 1 [file DataSheet_1.pdf]

Supplementary Material

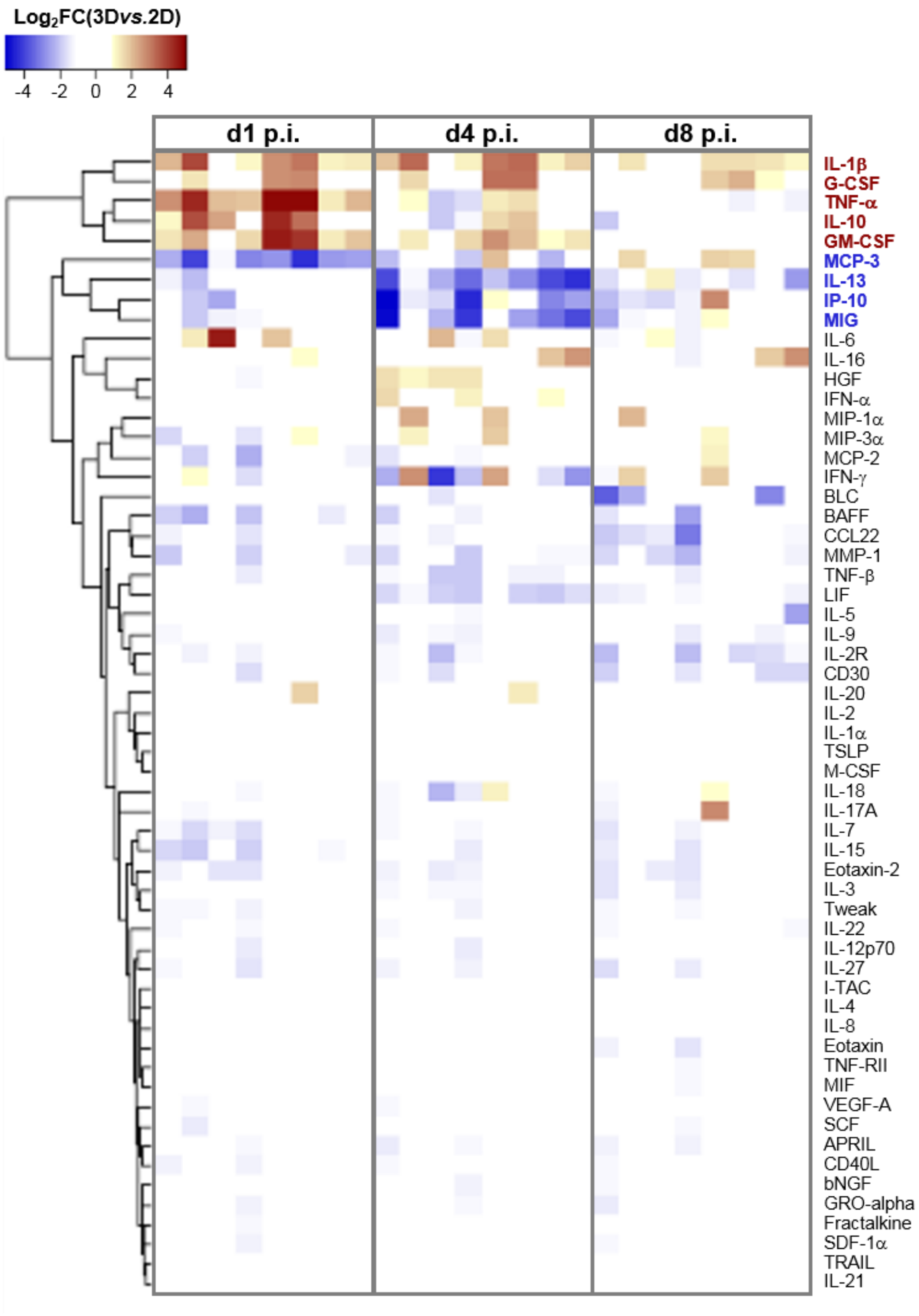

Supplementary Figure 1. Cytokines differentially secreted between the 2D and 3D *in vitro* granuloma models. Concentration of cytokines on days 1, 4 and 8 post-infection (p.i.) was quantified by multiplex bead-based immunoassay. Hierarchically clustered heat map representing log_2 of the fold change (FC) between the 3D and 2D models. Cytokines upregulated in the 3D model are depicted in red, while those downregulated are pictured in blue.

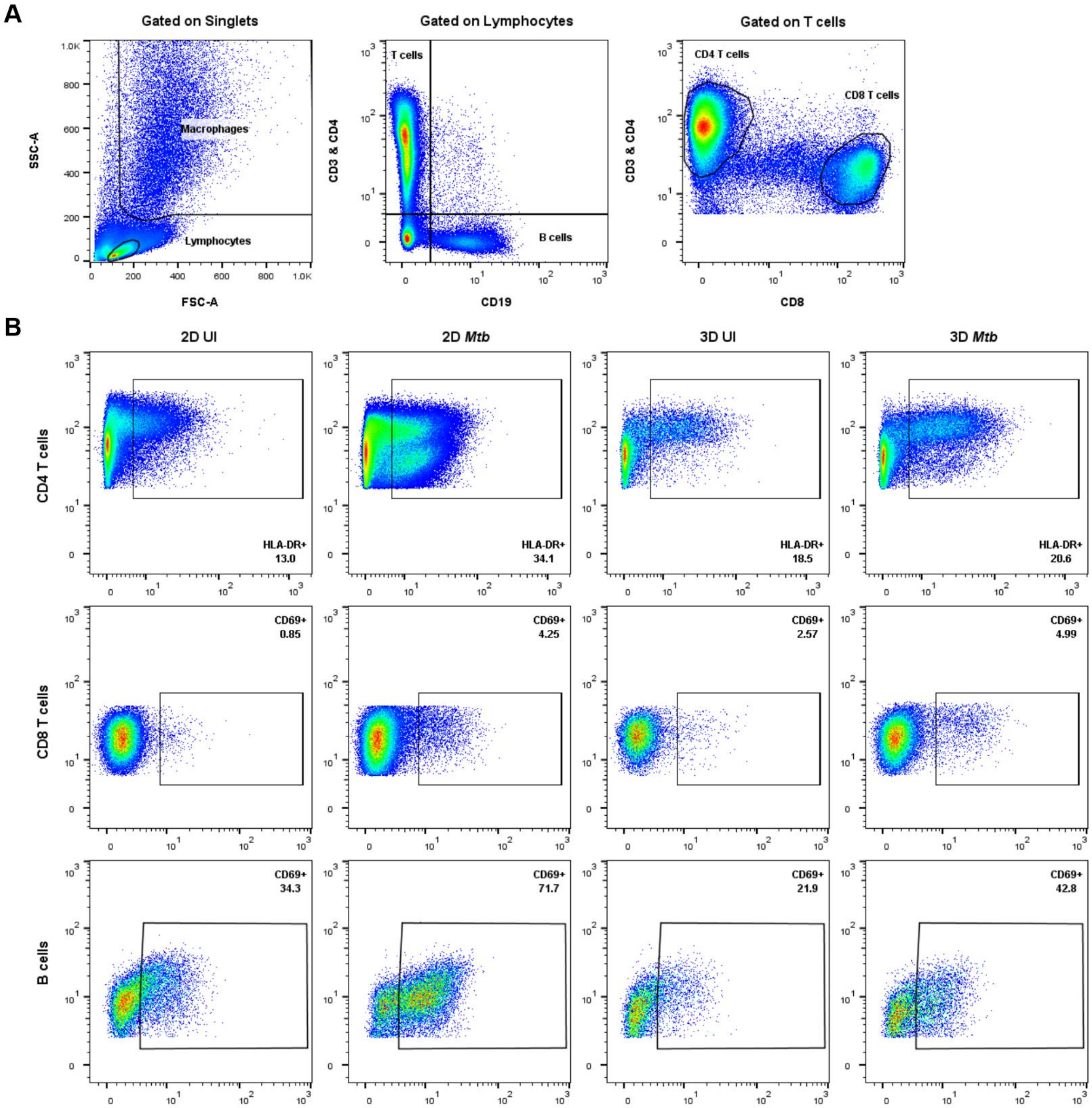

Supplementary Figure 2. Gating strategy for the analysis of lymphocyte activation by flow cytometry. (A) Lymphocytes were morphologically gated and the various subsets were identified by expression of specific surface markers. **(B)** Representative dotplots showing the acquisition of activation surface markers CD69 and HLA-DR in the 2D and 3D granuloma models.

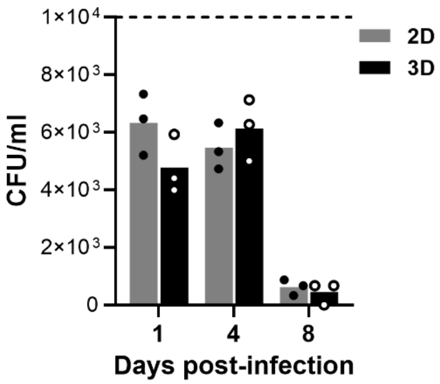

Supplementary Figure 3. Bacterial load in the absence of host cells. Bacterial load was quantified by colony forming unit (CFU) assessment. The bars represent the mean value and each dot the value of a technical replicate. The dashed line indicates the inoculum.

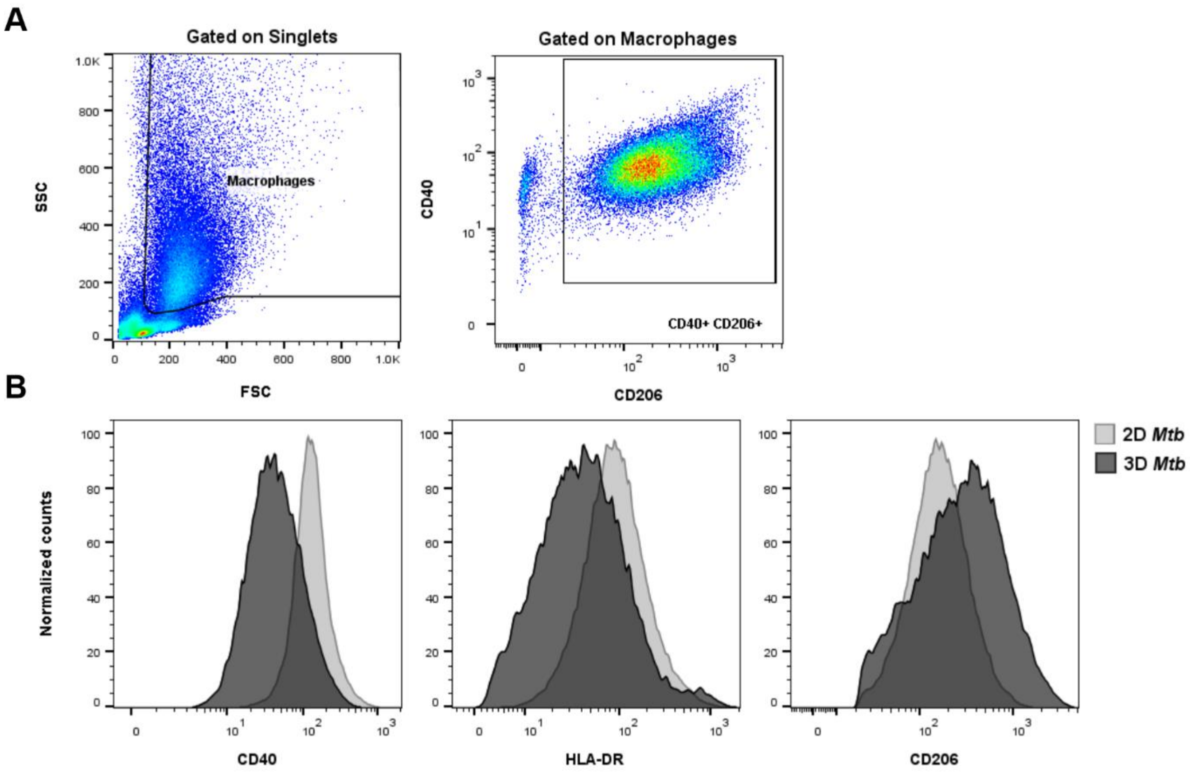

Supplementary Figure 4. Gating strategy for the analysis of macrophage polarization by flow cytometry. **(A)** Upon morphological gating, macrophages were identified by the expression of CD40 and CD206. **(B)** Representative histograms showing the expression levels of surface markers CD40, HLA-DR and CD206 in the 2D and 3D granuloma models.

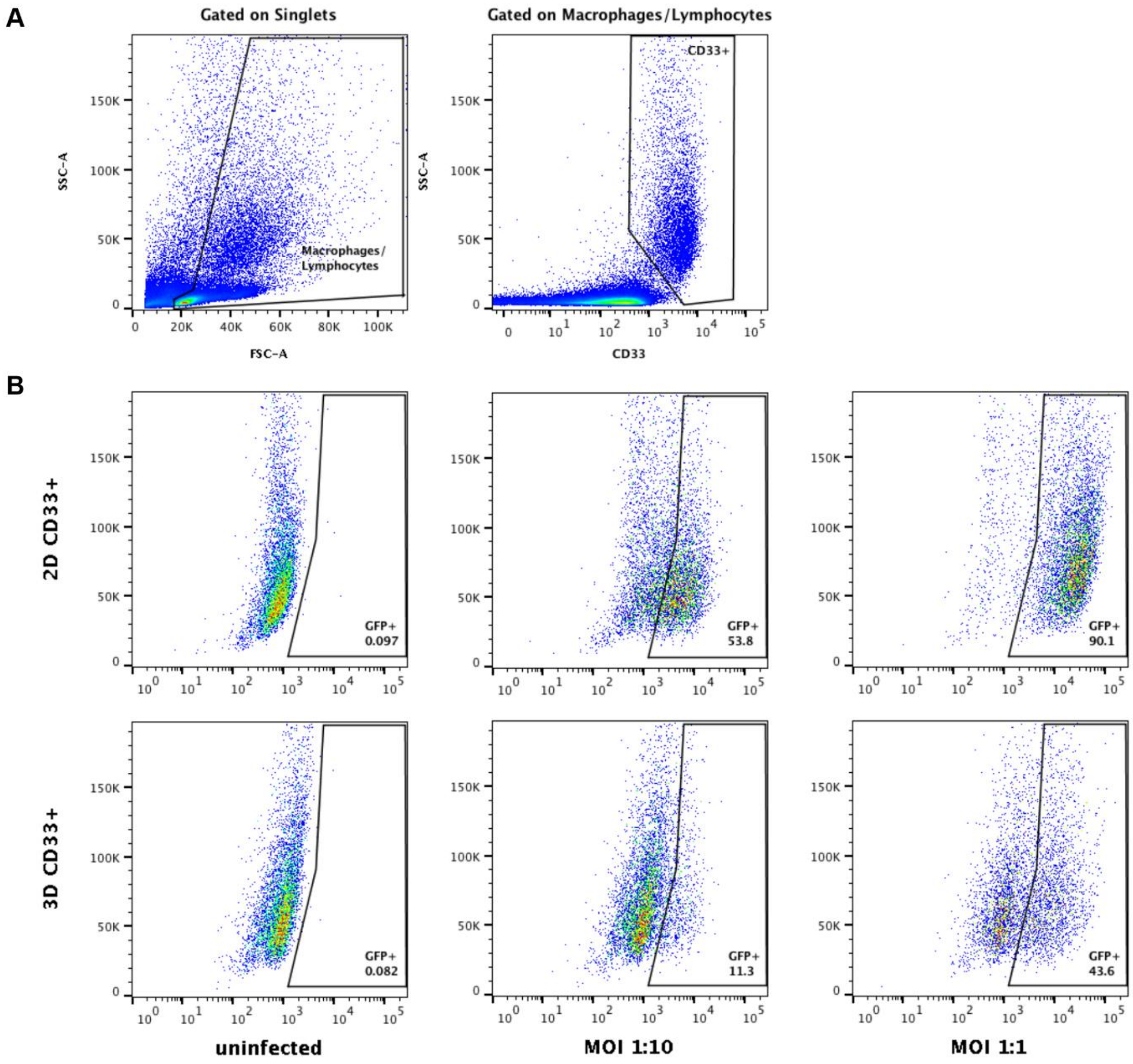

Supplementary Figure 5. Gating strategy for the analysis of phagocytosis by flow cytometry. **(A)** Upon morphological gating, macrophages were identified by the expression of CD33. **(B)** Representative dotplots showing the phagocytosis of *Mtb* H37Ra::GFP by macrophages recovered from 2D and 3D granuloma models.

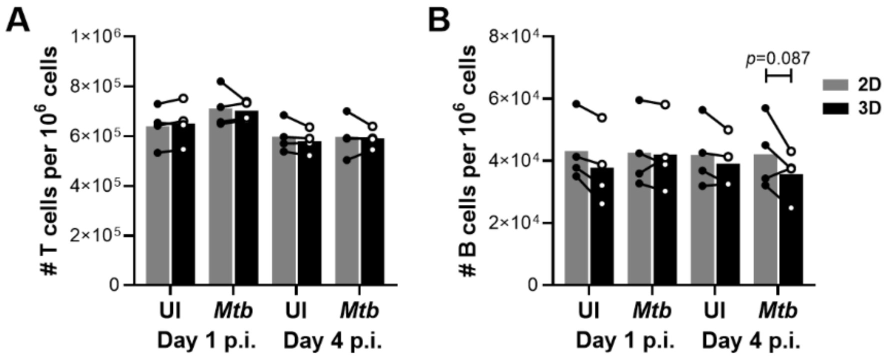

Supplementary Figure 6. Total number of retrieved T and B cell populations from 2D and 3D granulomas. Total number of T cells **(A)** and B cells **(B)** per million cells determined by flow cytometry. Upon morphological gating, lymphocyte subpopulations were identified by the expression of surface markers CD3 (T cells) and CD19 (B cells). Circles represent the value for each individual donor, lines connect results from the same donor, and bars indicate mean values of four donors. Statistical analysis was performed using two-way repeated measures ANOVA and Sidak's multiple comparisons test. None of the comparisons between the two models was significant.

Experiment		n=	Independent donors used																	
Granuloma formation	Fig. 1B-D	8		■	■	■		■	■			■		■		■				
Cytokine quantification	Fig. 2A-B, 3F	8		■	■	■		■	■			■		■		■				
Bacterial load	Fig. 3A	8		■	■	■		■	■			■		■		■				
Auramine/Nile red staining	Fig. 4A-B	8		■	■	■		■	■			■		■		■				
Flow cytometry	Fig. 2C, 3B+E	4				■			■					■		■				
Hypoxia	Fig. 4C	3					■										■	■		
Phagocytosis	Fig. 3C	4								■					■	■			■	
ROS production	Fig. 3D	3													■	■			■	
Exogenous GM-CSF	Fig. 3H	8	■	■		■		■	■		■		■							■
Hypoxia anti-TNF-α	Fig. 4D	3									■					■			■	

Supplementary Table 1. Overview of the independent PBMC donors used for the current study.
